# Assessing the International Transferability of a Machine Learning Model for Detecting Medication Error in the General Internal Medicine Clinic: Multicenter Preliminary Validation Study

**DOI:** 10.2196/23454

**Published:** 2021-01-27

**Authors:** Yen Po Harvey Chin, Wenyu Song, Chia En Lien, Chang Ho Yoon, Wei-Chen Wang, Jennifer Liu, Phung Anh Nguyen, Yi Ting Feng, Li Zhou, Yu Chuan Jack Li, David Westfall Bates

**Affiliations:** 1 Department of Biomedical Informatics Harvard Medical School Boston, MA United States; 2 College of Medical Science and Technology Graduate Institute of Biomedical Informatics Taipei Medical University Taipei City Taiwan; 3 Department of Medicine Brigham and Women's Hospital Harvard Medical School Boston, MA United States; 4 Doctor of Public Health Program Harvard TH Chan School of Public Health Boston, MA United States; 5 Department of Epidemiology Harvard TH Chan School of Public Health Boston, MA United States; 6 Department of Emergency Medicine Brigham and Women's Hospital Harvard Medical School Boston, MA United States; 7 International Center for Health Information Technology Taipei Medical University Taipei City Taiwan; 8 Division of General Internal Medicine and Primary Care Brigham and Women’s Hospital Harvard Medical School Boston, MA United States; 9 Graduate Institute of Biomedical Informatics College of Medical Science and Technology Taipei Medical University Taipei City Taiwan; 10 Department of Dermatology Taipei Municipal Wan Fang Hospital Taipei City Taiwan; 11 Clinical and Quality Analysis, Information Systems Partners HealthCare Somerville, MA United States

**Keywords:** electronic health records, patient safety, clinical decision support, medication alert systems, machine learning

## Abstract

**Background:**

Although most current medication error prevention systems are rule-based, these systems may result in alert fatigue because of poor accuracy. Previously, we had developed a machine learning (ML) model based on Taiwan’s local databases (TLD) to address this issue. However, the international transferability of this model is unclear.

**Objective:**

This study examines the international transferability of a machine learning model for detecting medication errors and whether the federated learning approach could further improve the accuracy of the model.

**Methods:**

The study cohort included 667,572 outpatient prescriptions from 2 large US academic medical centers. Our ML model was applied to build the original model (O model), the local model (L model), and the hybrid model (H model). The O model was built using the data of 1.34 billion outpatient prescriptions from TLD. A validation set with 8.98% (60,000/667,572) of the prescriptions was first randomly sampled, and the remaining 91.02% (607,572/667,572) of the prescriptions served as the local training set for the L model. With a federated learning approach, the H model used the association values with a higher frequency of co-occurrence among the O and L models. A testing set with 600 prescriptions was classified as *substantiated* and *unsubstantiated* by 2 independent physician reviewers and was then used to assess model performance.

**Results:**

The interrater agreement was significant in terms of classifying prescriptions as *substantiated* and *unsubstantiated* (κ=0.91; 95% CI 0.88 to 0.95). With thresholds ranging from 0.5 to 1.5, the alert accuracy ranged from 75%-78% for the O model, 76%-78% for the L model, and 79%-85% for the H model.

**Conclusions:**

Our ML model has good international transferability among US hospital data. Using the federated learning approach with local hospital data could further improve the accuracy of the model.

## Introduction

Medication errors are a major contributor to morbidity and mortality [1]. Although the exact number of deaths related to medical errors is still under debate, the *To Err Is Human* report estimated that the figure might be approximately 44,000 to 98,000 per year in the United States alone [2]. Medication errors also result in excess health care–related costs [3], which are estimated at more than US $20 billion per year in the United States. Preventable adverse drug events (ADEs) also appear to be common not only in the hospital but also in the ambulatory setting, with one estimate amounting to US $1.8 billion annually for treating them [4,5]. Reducing medication errors is crucial to enhance health care quality and improve patient safety. However, considering the time and cost needed, it is impossible for hospitals to double-check every prescription made by every physician in real time.

To combat this problem, studies have shown that health information technology (IT) presents a viable solution [6,7]. Among all IT tools, clinical decision support systems that can provide real-time alerts have demonstrated perhaps more effective in helping physicians to prevent medication errors [8-11]. However, the impact of these applications has been variable [12]. In addition, the vast majority of the currently deployed alert systems are rule based, which means that they have explicitly coded logic written to identify medication errors [13-15]. However, these rule-based systems are generally set to go off too frequently because of the lack of adaptability in clinical practice, leading to alert fatigue, which in turn can increase ADE rates [16-19].

Machine learning (ML) has shown promising results in medicine and health care [20-22], especially in relation to clinical documentation and prescription prediction [23-25]. Unsupervised learning, which is a type of ML algorithm used to establish relationships within data sets without labels, combined with a well-curated and large data set of prescriptions has the potential to generate algorithmic models to minimize prescription errors [26]. Previously, we had presented an ML model that evaluated whether a prescription was explicitly substantiated (by way of diagnosis or other medications) and prevented medication errors from occurring. The model was named as the appropriateness of prescription (AOP) model [27]. It contained disease-medication (D-M) associations and medication-medication (M-M) associations that were identified through unsupervised association rule learning. These associations were generated based on prescription data from Taiwan’s local databases (TLD), which had collected health information from nearly the entire Taiwanese population (about 23 million people) for over 20 years [28]. The AOP model has been validated in 5 Taiwanese hospitals and continues to have high accuracy (over 80%) and high sensitivity (80%-96%), highlighting the model’s potential to have a true clinical impact [29].

As physicians in Taiwan are educated with the same evidence-based guidelines as physicians in the United States, in theory, the experience-based ML model generated from TLD could be transferable to US clinical practice. However, there is no validation study that examines the transferability of the TLD-developed ML model in US health care systems. Although there are a few research studies demonstrating the feasibility of transferring ML models across health care institutions [30,31], one of the major challenges to the transferability of ML models in health care is that most of these models are trained using single-site data sets that may be insufficiently large or diverse [32]. Recently, federated learning has become an emerging technique to address the issues of isolated data islands and privacy, in which each distinct data federate trains their own model with their own data before all the federates aggregate their results [33]. In our study, we undertook a cross-national multicenter study to validate the performance of the AOP model in detecting the explicit substantiation of prescriptions using an enriched data set from the electronic health record (EHR) system of Brigham Women’s Hospital (BWH) and Massachusetts General Hospital (MGH). Both are Harvard Medical School teaching hospitals. To the best of our knowledge, this is the first cross-national multicenter study to examine the transferability of an ML model for the detection of medication errors. Detailed analyses were conducted to evaluate the effectiveness of the AOP model, and a federated learning approach was applied to explore the potential to construct a model with better performance using cross-national data sets.

## Methods

### Study Cohort

The study cohort comprised adult patients (aged ≥18 years) who had received any prescription (with at least one diagnosis and one medication) from clinicians affiliated to the Department of Internal Medicine at BWH or MGH during an outpatient clinical visit (the index visit) over 3 years, from January 1, 2017, to December 31, 2019. We extracted the data from the Partners HealthCare database, which has used an EPIC-based EHR system (Epic Systems Corporation) since 2016. No prescriptions were needed to be excluded because of missing values. We collected data such as demographic characteristics (age, sex, and ethnicity), diagnoses, problem lists, and prescribed medications. The age, sex, and ethnicity distributions within the BWH/MGH data set were as follows: age (years; mean 53.4, SD 19.8), sex (male 36% and female 64%), ethnicity (White 80%, Black 8%, Hispanic 7%, Asian 3%, Others 2%). The Partners Human Research Committee (Institutional Review Board protocol 2019P003566) approved this study’s protocol and design.

For deidentification, patient names and medical record numbers were removed from the data set, and a random study ID was assigned to each patient. A total of 667,572 prescriptions were included in the study. For data processing, we mapped the EPIC and HCPCS (Healthcare Common Procedure Coding System) medication coding systems to the RxNorm coding system and then mapped the RxNorm coding system to the Anatomical Therapeutic Chemical Classification System before we password-protected, encrypted, and sent the data to the AOP model. For prescriptions that were sampled to be evaluated by human physicians to determine the AOP model’s performance, additional clinical notes or office notes were requested to provide clinical context.

### Model Development

**Figure 1:**
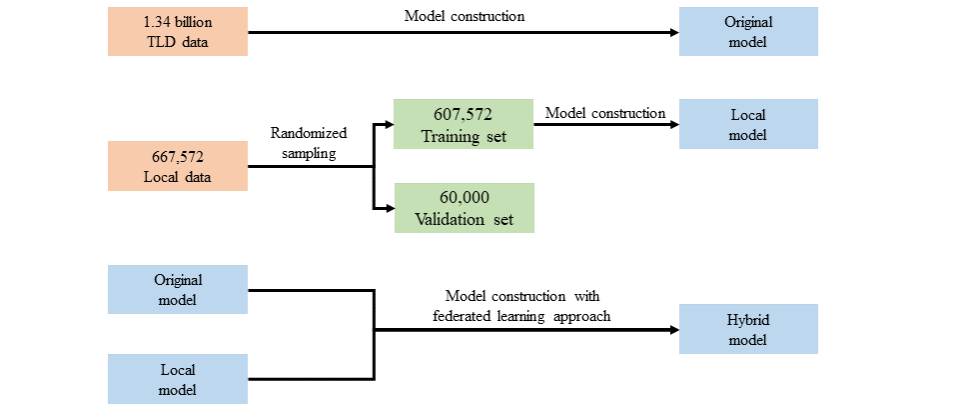
Research flowchart of the original model, local model, and hybrid model development. TLD: Taiwan's local databases.

**Figure 2:**
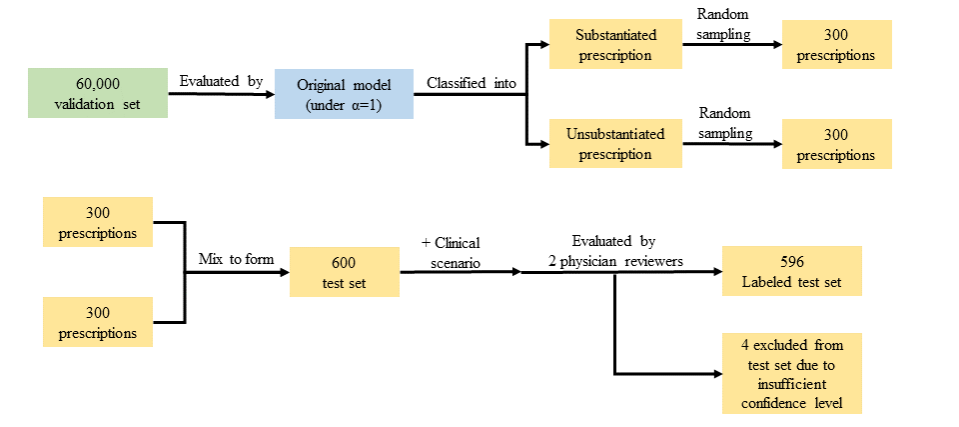
Research flowchart of the test set development.

A detailed flowchart of the study design is shown in Figures 1-2. The original model (O model) used in this study was constructed using the data of 1.93 billion outpatient prescriptions in the TLD from January 1, 2011, to December 31, 2015. The TLD, which contains data from over 25 million enrollees and covered over 99% of Taiwanese residents’ medical records, including cancer registry and mortality data [27]. Although the ethnicity data were not directly coded into TLD, based on the Taiwanese National Census data published in 2014 [34], over 97% of Taiwanese residents are of Asian ethnicity. The sex and age distributions of the TLD were as follows: age (years; mean 46.6, SD 23.3) and sex (male 45% and female 55%). Previous studies have validated the accuracy of diagnoses of major diseases in the TLD [35,36]. We excluded 590 million prescriptions for at least one of 2 reasons: (1) invalid or missing disease and/or medication codes and (2) prescriptions given by traditional Chinese medicine doctors. The remaining 1.34 billion prescriptions were used to generate the D-M and M-M associations. In summary, the data comprised 2.39 billion diagnoses coded in the International Classification of Disease v.10-Clinical Modification format and 4.14 billion medications coded according to the ATC classification system. We then applied the method described in our previous study to construct the AOP model [28]. In brief, the AOP model determined a prescription to be *substantiated* if each medication appearing in the prescription could be explained by a relevant disease and/or medications on the same prescription. However, if there were one or more medications in a prescription that could not be explained by any of the diagnoses within the same prescription, then the prescription would be viewed as *unsubstantiated*. The ratio between the joint probability of the D-M and the M-M associations was calculated as previously described (termed as the *Q value*) [27]. To develop a more sophisticated model that considers both age and sex, we calculated different Q values for different sex and age groups (5 years as an age group). To address the issue of pseudo association (eg, insulin may be explained by hypertension because hypertension and type 2 diabetes mellitus are common comorbidities), we only used the D-M association that had the highest Q value and discarded the Q values of the remaining D-M associations. The threshold value (α) was defined as 1 by default, which is commonly used in association rule mining studies [37]. If the Q value was greater than α, then the association was defined as a positive D-M or M-M association; if the Q value was less than α, then the association was defined as a negative D-M or M-M association. If both the D-M and M-M associations were positive with respect to a single prescription, then only our model considered a prescription to have been substantiated.

To construct the local model (L model), a validation set with 8.98% (60,000/667,572) of the prescriptions was first randomly sampled to form a validation set, and the remaining 91.02% (607,572/667,572) of the prescriptions served as the training set. We then applied the same abovementioned method to construct the L model with the training set (Figure 1). Using a federated learning approach, we assessed the Q values from both the O and L models. If a D-M or M-M association was observed in both the O and the L models, then we selected the Q values with a higher frequency of co-occurrence between the 2 models to ultimately develop the hybrid model (H model).

### Test Set Development

To establish the final test set, we first used the O model (with α=1) to evaluate the validation set (Figure 2), which resulted in the classification of a group of substantiated prescriptions and a group of unsubstantiated prescription groups. We randomly sampled 300 prescriptions from each group and then combined them with their respective clinical scenarios (based on the clinical note of the same visit when the prescription was prescribed) to form an enriched test set to ensure that there would be sufficient numbers of unsubstantiated prescriptions for further analysis. Two licensed physicians, blinded to the percentage of model-determined substantiated or unsubstantiated prescriptions within the test set, independently examined each set of these randomly sampled prescriptions. The severity of each unsubstantiated prescription was further classified as potentially life-threatening, serious, or significant following the definitions as previously described [38]. A life-threatening, unsubstantiated prescription was defined as the potential to cause symptoms that, if left untreated, would put the patient at risk for death. A serious, unsubstantiated prescription was defined as there is the potential to cause symptoms associated with a severe level of harm but not great enough to be considered life-threatening. A significant, unsubstantiated prescription was defined as there is the potential to cause symptoms that, although harmful to the patient, pose little or no threat to the patient’s functional status. Quality checks were performed throughout the study period by reviewing the physician reviewers’ responses to each set of randomly sampled prescriptions, as described above. In each of these prescriptions, there may have existed one or several medications that led to the judgment of an *unsubstantiated prescription*. We asked the physician reviewers to highlight the problematic medications within a prescription. Tables 1 and 2 display a sample of reviewer-determined substantiated or unsubstantiated prescriptions from the final test set, with problematic medications highlighted in red. To evaluate the physicians’ confidence regarding their classification of adequate substantiation and the severity of potential adverse effects, we asked them to rate their decisions on a 6-point scale, as described previously [4]. We excluded the prescription if one of the physicians rated their confidence level lower than 4 (ie, corresponding to a confidence level <50%). Any differences between the 2 physician reviewers’ judgments about the classification of substantiation and severity of potential adverse effects were resolved by discussion. If a discussion was insufficient to resolve the problem, then a senior physician was consulted and the final decision was made. Through this entire process, we generated the *ground truths* for whether each of these 600 prescriptions was explicitly substantiated by a declared diagnosis and/or other medications.

**Table 1:** An example of a substantiated prescription as determined by physician reviewers. The patient was a 74-year-old woman with a history of rheumatoid arthritis, hypertension, and moderate aortic stenosis, who presented with shortness of breath that had become worse than 1 year ago, and for whom ankle edema had been noted in the last couple of weeks.

Code		Disease and medication name
**ICD-10-CM^a^ code**		
	I35.0	Nonrheumatic aortic (valve) stenosis
	I10	Hypertensive disorder
	I 73.0	Raynaud’s disease
	E78.5	Hyperlipidemia
**ATC^b^ code**		
	B01AC06	Aspirin
	C10AA05	Atorvastatin
	C03CA01	Furosemide
	C09CA01	Losartan

^a^ICD-10-CM: International Classification of Disease-10-Clinical Modification

^b^ATC: Anatomical Therapeutic Chemical.

**Table 2:** An example of unsubstantiated prescription as determined by physician reviewers. The patient was a 76-year-old man who presented with an unsteady gait and for management of his anticonvulsant medications.

Code		Disease and medication name
**ICD-10-CM^a^ code**		
	R26.9	Unspecified abnormalities of gait and mobility
	G40.309	Generalized idiopathic epilepsy and epileptic syndromes, not intractable, without status epilepticus
**ATC^b^ code**		
	C10AA01	*Simvastatin* ^c^
	L01BA01	*Methotrexate sodium*
	N03AX14	Levetiracetam
	A02BC01	*Omeprazole*
	B01AA03	*Jantoven*
	P01BA02	*Hydroxycholoroquine*
	B03BB01	*Folic acid*

^a^ICD-10-CM: International Classification of Disease-10-Clinical Modification

^b^ATC code: Anatomical Therapeutic Chemical code.

^c^Medications that could not be explained by the patient’s listed diagnoses were italicized.

### Evaluation

To compare the performances of the O, L, and H models, the performance of each model on the final test set was measured using sensitivity, specificity, negative predictive value (NPV), positive predictive value (PPV; positive=unsubstantiated prescription), and accuracy. To examine the effect of α on model performance, we adjusted α from .5 to 1.5 (ie, α∈[.5; 1.5]).

### Statistical Analysis

We used a 2-tailed Student *t* test for measuring continuous variables with a normal distribution and presented the results as mean (SD). The chi-square test was used to compare categorical data, and the results were presented as counts and percentages. For data with skewed distributions, we computed their median and IQR values and used the Wilcoxon rank-sum test for comparison [39]. The Cohen kappa coefficient (κ) statistic was applied to measure the interrater agreement of physicians on whether prescriptions were substantiated. Statistical analyses were performed using R version 3.6.2 [40].

## Results

The interrater agreement for the substantiation (or not) of prescriptions for the test set was high (κ=0.92; 95% CI 0.89 to 0.95). With substantiated prescriptions, the agreement was also good for assessing severity (κ=0.84; 95% CI 0.73 to 0.95). In total, 4 prescriptions were excluded from the test set because of insufficient physician-reviewer confidence levels (scores lower than 3). Among the remaining 596 prescriptions, 232 prescriptions were determined to be unsubstantiated and 364 prescriptions were deemed substantiated. No unsubstantiated prescription was judged to be life-threatening. Among the 232 unsubstantiated prescriptions, 27 (11.6%) prescriptions were found to be associated with serious potential ADEs and 205 (88.4%) were determined to be associated with significant potential ADEs.

The performances of the O, L, and H models with different thresholds (ranging between 0.5 and 1.5) are shown in Table 3. For the O model under different thresholds, the sensitivity ranged from 82% to 92%, the specificity ranged from 70% to 76%, PPV ranged from 66% to 68%, NPV ranged from 83% to 92%, and accuracy ranged from 75% to 78%. For the L model at different thresholds, the sensitivity ranged from 76% to 85%, the specificity ranged from 73% to 76%, PPV ranged from 67% to 68%, NPV ranged from 70% to 80%, and accuracy ranged from 76% to 78%. For the H model with different thresholds, the sensitivity ranged from 56% to 79%, the specificity ranged from 87% to 93%, PPV ranged from 80% to 85%, NPV ranged from 74% to 86%, and accuracy ranged from 79% to 85%.

**Table 3:** Performance comparison between different models under different threshold values (α) based on 596 physician-validated cases of ground truth.

Threshold value (α)^a^	O Model^b^					L Model^c^					H Model^d^				
	Sen^e^	Spe^f^	PPV^g^	NPV^h^	Accu^i^	Sen	Spe	PPV	NPV	Accu	Sen	Spe	PPV	NPV	Accu
1.5	0.92	0.70	0.66	0.83	0.75	0.85	0.73	0.67	0.88	0.78	0.79	0.87	0.80	0.87	0.84
1.4	0.91	0.71	0.66	0.92	0.78	0.83	0.74	0.67	0.87	0.77	0.79	0.88	0.80	0.86	0.84
1.3	0.90	0.71	0.67	0.92	0.79	0.83	0.74	0.67	0.87	0.77	0.79	0.88	0.81	0.87	0.85
1.2	0.90	0.71	0.67	0.92	0.79	0.82	0.74	0.67	0.87	0.77	0.78	0.90	0.83	0.86	0.85
1.1	0.89	0.73	0.68	0.87	0.78	0.82	0.75	0.68	0.87	0.78	0.78	0.90	0.84	0.86	0.85
1.0	0.88	0.74	0.68	0.90	0.79	0.81	0.75	0.67	0.86	0.77	0.76	0.91	0.84	0.86	0.85
0.9	0.88	0.74	0.68	0.90	0.79	0.80	0.75	0.67	0.85	0.77	0.74	0.91	0.85	0.71	0.84
0.8	0.86	0.74	0.68	0.89	0.79	0.78	0.76	0.67	0.84	0.77	0.70	0.92	0.85	0.83	0.83
0.7	0.84	0.75	0.68	0.88	0.78	0.77	0.76	0.68	0.84	0.77	0.65	0.92	0.85	0.81	0.82
0.6	0.83	0.75	0.68	0.87	0.78	0.76	0.76	0.68	0.83	0.76	0.61	0.93	0.84	0.79	0.80
0.5	0.82	0.76	0.68	0.87	0.78	0.76	0.76	0.67	0.83	0.76	0.56	0.93	0.84	0.77	0.79

^a^The ratio between the joint probability of the disease-medication (D-M) and the medication-medication (M-M) associations were calculated as previously described in the Methods (termed the *Q value*). If the Q value was greater than α, then this association was defined as a positive disease-medication (D-M) or medication-medication (M-M) association. However, if the Q value was less than α, then this association was defined as a negative D-M or M-M association. Our model considered a prescription to have been substantiated only if both the D-M and M-M associations were positive with respect to a single prescription.

^b^O model: original model.

^c^L model: local model.

^d^H model: hybrid model.

^e^Sen: sensitivity.

^f^Spe: specificity.

^g^PPV: positive predictive value.

^h^NPV: negative predictive value.

^i^Accu: accuracy.

A comparison of the substantiated prescription and unsubstantiated prescription groups, as determined by the physician reviewers, is summarized in Table 4. The average ages (SD) in the substantiated prescription group and the unsubstantiated prescription group were 70.3 years (SD 12.7) and 68.1 years (SD 14.2), respectively. None of the patient characteristics (ie, sex, age) were significantly associated with unsubstantiated prescriptions (*P*=.72 and *P*=.05, respectively). The substantiated prescription group had a higher number of diagnoses than the unsubstantiated group (median 3 [IQR 3] vs median 2 [IQR 3]; *P*<.001). In contrast, the unsubstantiated prescription group had higher numbers of medications than the substantiated group (median 2 [IQR 1] vs median 3 [IQR 4.75]; *P*<.001).

**Table 4:** Comparison of patient characteristics between the substantiated and unsubstantiated prescription groups.

Characteristics	Substantiated prescriptions	Unsubstantiated prescriptions	*P* value
Sex (male/female)	249/115	156/76	.72
Age (years), mean (SD)	70.3 (12.7)	68.1 (14.2)	.05
Number of diagnoses, median (IQR)	3 (3)	2 (3)	<.001
Number of medications, median (IQR)	2 (1)	3 (4.75)	<.001

In total, 32 medication classes appeared in the unsubstantiated prescription group. The top 7 medication classes most frequently associated with unsubstantiated prescriptions, categorized into potential severity classes (serious and significant), are shown in Table 5. In general, the most frequent medication classes were opioid analgesic (n=34), benzodiazepine (BZD; n=27), selective serotonin reuptake inhibitor (SSRI; n=17), nonopioid analgesic (n=16), proton pump inhibitor (PPI; n=15), antihistamine (n=14), and anticoagulant (n=13). For the serious severity class, the most frequent medication classes were opioid analgesic (n=20), BZD (n=6), anticoagulant (n=5), β-blocker (n=4), angiotensin-converting enzyme inhibitor/angiotensin II receptor blocker (n=4), antipsychotic (n=3), and anticholinergic (n=3). As for the significant severity class, the most frequent medication classes were BZD (n=21), SSRI (n=16), PPI (n=15), and opioid analgesic (n=14).

Under α=1, 11.6% (27/232) of the cases from the unsubstantiated prescription group, which were determined as unsubstantiated by the O model (true positive), were determined as substantiated by the H model (false negative). Among these cases, opioid analgesic (n=9) was the most common medication class. In contrast, 17.0% (62/232) of thecases from the substantiated prescription group, which were determined as unsubstantiated by the O model (false positive), were then determined as unsubstantiated by the H model (true negative). Opioid analgesic (n=18) was the most common medication class in these cases.

**Table 5:** The top 7 medication classes most frequently associated with unsubstantiated prescriptions as determined by physician reviewers are shown across the different classes of severity. There were no unsubstantiated prescriptions that were considered to be life-threatening in our study.

Medication class		Times each medication class appears, n
**Total**		
	Opioid analgesic	34
	BZD^a^	27
	SSRI^b^	17
	Nonopioid analgesic	16
	PPI^c^	15
	Antihistamine	14
	Anticoagulant	13
**Serious^d^**		
	Opioid analgesic	20
	BZD	6
	Anticoagulant	5
	β-blocker	4
	ACEi/ARB^e^	4
	Antipsychotic	3
	Anticholinergic	3
**Significant^f^**		
	BZD	21
	SSRI	16
	PPI	15
	Opioid analgesic	14
	Anticonvulsant	12
	Antihistamine	12
	Nonopioid analgesic	11

^a^BZD: benzodiazepine.

^b^SSRI: selective serotonin reuptake inhibitor.

^c^PPI: proton pump inhibitor.

^d^A serious, unsubstantiated prescription was defined as having the potential to cause symptoms associated with a severe level of harm but not great enough to be considered life-threatening.

^e^ACEi/ARB: angiotensin-converting enzyme inhibitor/angiotensin II receptor blocker.

^f^A significant, unsubstantiated prescription was defined as having the potential to cause symptoms that, while harmful to the patient, pose little or no threat to the patient’s functional status.

## Discussion

### Principal Findings

We evaluated the performance of the AOP ML model, developed in Taiwan, in determining whether prescriptions have been explicitly substantiated using EHR data from 2 large US academic hospitals. We found that the model performed well and that a hybrid learning approach had a higher accuracy than the individual model under most thresholds, exhibiting better specificity and NPV. This result indicates that additional efforts to retrain the model with training data from the local health care system holds promise in further improving the performance of the AOP model.

With TLD, researchers have identified several significant associations with high clinical impact, such as the association between nucleoside analogs and the risk of post liver resection hepatocellular carcinoma recurrence, and risk factors for poststroke dementia [41-43]. The thesis of the AOP model is that prescriptions solely comprising common D-M combinations in a large database, such as TLD, have a higher possibility of being substantiated. In contrast, medications less frequently prescribed for a given disease are more likely to be unsubstantiated. Although physicians in Taiwan are educated and trained with US guidelines, there are some differences in clinical practice between the 2 health care systems.

Therefore, a validation study is necessary to assess the transferability of such an ML model. Nowadays, research focusing on externally validating a health care ML model is rarely conducted [32], which is partly because of the expectation of poor transferability of complex ML models [44]. The overall results in this study showed a reasonable accuracy (78%-76% for the O model and 85%-79% for the H model), which demonstrated that the AOP model has the potential to be transferrable among the US clinical data sets. In this study, we found that the H model had the highest accuracy, which might be due to the fact that the O model was trained with sufficient amount of data so as to allow the supplementation of the performance of the L model to achieve better performance. To the best of our knowledge, this is the first multicenter study to specifically address the issue of international transferability of an ML model for the detection of medication errors, which can pave the path for other validation studies of this kind.

Alert fatigue can potentially cause physicians to ignore important clinical alerts, which lead to unwanted medication errors. Alert fatigue occurs if there is a high frequency of nonactionable and false alarms [8]. Most of the current CPOE (computerized physician order entry) systems use rule-based alerts to support clinical decision making. However, previous research has shown high *overridden alert* rates to rule-based alerts within the EMR, ranging from 49% to 96% [45]. ML-based approaches, which generate an alert based on past real-world prescribing behaviors extracted from a large database, appear to be an attractive approach to address alert fatigue and improve patient safety. Previous researchers have explored the feasibility of using an ML-based outlier detection system to detect medication errors. They found that three-fourth of the alerts generated by the system were determined to be valid based on 300 chart review results, after the modified algorithm model was created with data from 373,993 patients [26]. We applied a different ML approach and used a different database with more training data (over 1.3 billion) to construct our model, and our results were comparable. Another recent study estimated that an ML-based system could potentially save US $1.3 million in an outpatient setting through the prevention of adverse events, hinting at additional economic benefits that such systems may offer [46].

Among unsubstantiated prescriptions, 11.6% were found to be associated with potential ADEs, a finding that is similar to the number reported by Gandhi et al (13%) [4]. We found that patient characteristics were not significantly associated with unsubstantiated prescriptions, which suggests that the strategy to improve the prescription process for all patients may be more effective than focusing on specific patient subgroups. Interestingly, a similar finding was also demonstrated in a study of hospitalized patients [47]. In this study, we showed that higher numbers of medications were found to be significantly associated with unsubstantiated prescriptions than with substantiated prescriptions. Polypharmacy has long been a significant issue among older adults and is a known risk factor for adverse medical outcomes [48]. Although currently there are tools to assist in the identification of potentially inappropriate medications, such as the Screening Tool of Older People’s Prescriptions and the Screening Tool to Alert to Right Treatment criteria, no single tool has been shown to be sufficient in reducing the risk of unnecessary polypharmacy—it is likely that a combination of approaches may work best [49]. Furthermore, these criteria require physicians to make separate calculations, which might add additional cognitive burden and disrupt the clinical workflow.

Our model shows the potential to automatically identify unsubstantiated medications when a physician updates the patient’s active problem list, which can assist with the deprescribing process and potentially reduce pill burden. We further investigated which medication classes were most frequently associated with unsubstantiated prescriptions, and the opioid analgesics ranked the highest. It is worth noting that opioid analgesics also ranked as the top medication in prescriptions when predictions differed between the O and the H model, which reflects the different prescribing behaviors with respect to opioid analgesics between Taiwan and the United States. Clinical decision support tools could potentially play a role in actively managing opioid prescription behavior and provide the correct guidance [50]. Our study processed the data extracted from the EPIC-supported CPOE system, and successfully generated validation results. As EPIC is currently being used in multiple large US health care systems, it shows that our AOP model, while originally developed based on the TLD, may be applied in the US clinical environment. We envision that the AOP model will be integrated with the current CPOE system as an application to fire alerts on potentially inappropriate prescriptions in real time once physician prescribers complete their prescription in the system. If this model is validated with unenriched clinical data for use in clinical practice, then we also foresee that such an application may be able to suggest a list of recommended diagnoses for an unsubstantiated medication; alternatively, such an application may help to prompt physician prescribers to address potential medication errors (eg, medications attributed to the wrong patient). Another potential application would be to automatically facilitate medical record completeness during the error-prone medication reconciliation process [51].

This study has several limitations. First, even though we performed random sampling when we constructed the test set, it is possible that the selected prescriptions may present some bias because of a relatively small sample size (600 prescriptions), which might also explain why there were no unsubstantiated, physician-determined, life-threatening prescriptions in the test set. We did not apply common ML evaluation methods such as cross-validation or bootstrapping because of limited labeled data. However, considering the time and effort needed by a physician to evaluate whether a prescription was explicitly substantiated, we believe that using randomized sampling to construct a test set of 600 prescriptions was a reasonable approach for a preliminary model validation study. As the incidence of prescribing error was reported to be approximately 1%-2% [52], we used randomized sampling to construct an enriched, balanced test set to ensure that there were sufficient unsubstantiated prescriptions included for further analysis. Although using an enriched test set might lead to an overestimation of the model performance, this study is a critical step for preliminary AOP model validation, and we plan to validate our model in less enriched, more real-world data sets in the near future. The current AOP model only considered the patient’s sex, age, diagnoses, and medications. However, patients’ lab data and chief complaints may also impact prescribing behavior. We also did not compare the performance of the AOP model with the legacy rule-based alert systems built into the current EHR to confirm the value added by our model. The current AOP model did not consider dose-dependent errors. However, this issue is unlikely to undermine the value of the AOP model because identifying a dose-dependent error is a relatively straightforward rule-based question, and most of the current CPOE systems have built-in alert systems for detecting dose-dependent error [53,54]. It is worth noting that although our models’ sensitivities were good but not perfect, most medication error alert systems in use today are not designed to identify potential medication errors originating from D-M mismatch. In addition, our physician reviewers determined the severity of unsubstantiated prescriptions based on the prescribed medications instead of observing the ADEs in a real-world setting. It is possible that medication with the potential to cause serious ADE did not cause a serious event (eg, due to noncompliance). In this study, we only evaluated outpatient data from one specialty. Further work is needed to assess the AOP model’s performance prospectively in an inpatient setting and across different medical specialties to determine its actual impact on drug-prescribing behaviors. Finally, we constructed a federated learning model based on a data set with a predominantly Asian population (Taiwanese) and a data set with US patients, who had considerable differences in ethnic proportions. Further studies will be required to explore the contribution of ethnicity in the model’s predictive performance.

### Conclusions

In this preliminary study, we found that the AOP ML model based on TLD had good transferability with US prescription data in an outpatient setting. We also found that a model built with a federated learning approach, which combined models developed from TLD data and US local data, could further improve its accuracy as compared with models developed from each individual data set. This type of ML approach holds promise in improving alert fatigue, which has often been a major issue in traditional, rule-based alert systems.

## Abbreviations

ADE: adverse drug event
AOP: appropriateness of prescription
BWH: Brigham and Women’s Hospital
BZD: benzodiazepines
EHR: electronic health record
H model: hybrid model
IT: information technology
L model: local model
MGH: Massachusetts General Hospital
ML: machine learning
MOE: Ministry of Education
MOST: Ministry of Science and Technology
NPV: negative predictive value
O model: original model
PPI: proton pump inhibitor
PPV: positive predictive value
SSRI: selective serotonin reuptake inhibitors
TLD: Taiwan's local databases
